# The Book World of Medicine and Science

**Published:** 1906-12-22

**Authors:** 


### The Book World of Medicine and Science.

How to Find and Name Wild Flowers. By Thomas Fox, F.L.S., with an introduction by F. E. Hume, F.L.S. (London: Cassell and Company, Limited, 1s. 6d.)

This is an excellent handbook by which upwards of 1,200 species of flowering plants in the British Isles can be identified. A great deal has been written and said recently concerning the functions of museums as places of recreation and profitable study. This book shows how in field and by hedgerow one can become a worker in the greatest of all recreation museums—the museum of nature. This is a handbook for helping the study and interpretation of nature. It is catalogued and arranged. The multifarious specimens of the vegetable world which present themselves everywhere and at all seasons to the inquiring eye are here adequately and simply classified. The book forms a complete and ready reference to the museum of nature both for serious and casual student alike, to be carried in country walks and consulted in quiet commune with the beauties of the field. The classification of the flowers into the months at which they appear and under their natural colours enables the most unaccustomed observer to name and classify scientifically almost any plant that grows in the British Islands. If this little work does not succeed in stimulating an interest in advanced botanical studies, the fault is not due to the beauty or utility of the work.

The Quiet Hour. Selected by J. E. and H. S. (London, Simpkin Marshall and Company, price 1s.).

This little book has been designed by Mr. J. Ellis, the compiler, as one suitable to place on the waiting-room table of consultants, for the use of patient-visitors. The idea is, that the cheerful and helpful thoughts, by a multitude of writers, which are here brought together in an attractive form, cannot fail to be of service by diverting the thoughts of the patients, and helping them to pass the time of waiting pleasantly and profitably. The book is beautifully printed and most attractive to the eye. In the rush and hurry of everyday life it is not easy to find time for rest and quiet. This little book, by placing before the reader thoughts that will help and inspire, should therefore fulfil a very useful purpose. We can confidently recommend it to the attention of members of the medical profession who will find it an attractive addition to the table of their consulting-rooms. It is a book, too, which is especially suitable for convalscents, and should so become popular as a present.

Practical Advertising, 1906-7. (London: Mather and Crowther, Ltd.)

We have received the 1906-7 issue of this very useful work, which contains some excellent illustrations of modern advertisements and a most interesting article entitled "The Lessons of Sixty Years," dealing with the developments constantly being effected in the business of advertising. This is supplemented by very complete lists of newspapers and periodicals published throughout the British Empire, with important particulars regarding the price, circulation, date of publication, advertisement rates, etc., set forth very clearly and concisely. An admirable index renders the work of the greatest value to all interested in or connected with advertising and to business men generally.

